# Effects of a 30-min rest with a nap chair on task performance, sleepiness, and neurophysiological measures in men with suspected brain fatigue: a randomized controlled crossover trial

**DOI:** 10.3389/frsle.2024.1361886

**Published:** 2024-10-25

**Authors:** Minoru Fujino, Mikio Inoue, Yoshiharu Sonoda, Suminori Kono, Chikako Wakana, Shiro Mawatari, Takehiko Fujino

**Affiliations:** ^1^BOOCS Clinic Fukuoka, Fukuoka, Japan; ^2^Toyota Motor Corporation, Advanced Mobility System Development Div., Toyota, Japan; ^3^MedStat Corporation, Fukuoka, Japan; ^4^Institute of Rheological Functions of Food, Fukuoka, Japan

**Keywords:** short nap, nap chair, sleepiness, parasympathetic activation, brain fatigue

## Abstract

**Background:**

It has been suggested that a short nap in the afternoon may improve sleepiness, alertness, and task performance. The present study evaluated the effects of a 30-min rest with a new nap chair on task performance, sleepiness, and neurophysiological measures.

**Methods:**

A randomized controlled crossover trial with a 1-week interval was carried out at the BOOCS Clinic Fukuoka in Japan. The subjects were male workers aged 20 to 64 years with suspected brain fatigue, which was defined by the Profile of Mood Status 2. The intervention was a 30-min rest with an office chair or a nap chair. The primary outcome was the performance in the Uchida-Kraepelin test. The secondary outcomes included the Karolinska Sleepiness Scale and 15-min heart rate variability (HRV). The changes after the nap-chair rest and office-chair rest were compared. Repeated measures analysis of variance with nesting was used in the statistical analysis.

**Results:**

Twenty participants were eligible and entered the crossover trial. The overall 15-min score in the Uchida-Kraepelin test improved after the nap-chair rest and after the office-chair rest to almost the same extent (5.9 vs. 5.5 points, *P* = 0.68). The Karolinska Sleepiness score significantly decreased after the nap-chair rest, and the between-treatment difference in the decrease was highly significant (*P* = 0.0004). The average duration of sleep during rest was prominently longer in the nap-chair rest than in the office-chair rest (19.0 vs. 7.6 min, *P* = 0.002). No participants experienced REM sleep during the rest. LF and HF powers of the HRV were greater during the nap-chair rest than during the office-chair rest, the difference in the HF power being substantial.

**Conclusion:**

A 30-min rest with the nap chair did not appreciably improve the performance in the Uchida-Kraepelin test as compared with the office-chair rest. The nap-chair rest induced a substantially longer sleep accompanied with a parasympathetic activation, thereby resulting in a material improvement in sleepiness after the rest.

## Introduction

Napping has long been recommended to maintain alertness and job performance for those with occupational sleep deprivation such as shift workers and long-distance drivers (Rosekind et al., 1995; Takahashi, 2003). It has been suggested that a short nap in the afternoon may improve sleepiness, alertness, and task performance among those with normal nocturnal sleep (Takahashi, 2003; Milner and Cote, 2009; Dutheil et al., 2021). The timing and duration of an afternoon nap have been of particular interest in relation to the effects of napping. It seems to be desirable to take a nap of < 30 min in the early afternoon to avoid prolonged sleep latency and sleep inertia (Takahashi, 2003; Milner and Cote, 2009). In a series of trials conducted with university students (Hayashi et al., 1999a,b; Hayashi and Hori, 1998), the investigators reported that a 20-min nap taken soon after lunch (12:20 and thereafter) improved sleepiness, subjective work performance, and arousal levels as measured by brain alpha wave (Hayashi et al., 1999a,b) and that a 20-minute nap taken slightly later (14:00 and thereafter) additionally improved objective work performance (Hayashi and Hori, 1998). In these studies (Hayashi et al., 1999a,b; Hayashi and Hori, 1998), a computer device was used to assess the task performance with respect to logical reasoning, calculation (addition), visual detection, and auditory vigilance.

The effects of napping on the autonomic nervous system have also been a matter of interest. The heart rate variability (HRV) parameters are used for the assessment of sympathetic and parasympathetic nerve activity (Trinder et al., 2012; Chen et al., 2021). Nocturnal sleep is known to affect the HRV parameters, depending on sleep stages (Trinder et al., 2012). The elevation of the parasympathetic nerve activity during nocturnal sleep is considered to be beneficial for the cardiovascular system (Trinder et al., 2012). Few studies have examined the autonomic nerve activity during a nap (Cellini et al., 2016; Chen et al., 2021).

It is thus practically important to take a nap efficiently in the work environment. Previously, chairs for napping were designed mostly focusing on the relaxing angles of the chair, as reviewed elsewhere (Oyama et al., 2019; Nishida et al., 2022). Light and noise are important factors disturbing the induction and maintenance of sleep. A team of the Toyota Motor Cooperation has developed a nap chair equipped with functions of controlling light, noise, and temperature as well as relaxing angle. The present study evaluated the effects of a 30-min rest with the nap chair on task performance by means of the Uchida-Kraepelin test, sleepiness, and HRV in a crossover trial of working men with suspected brain fatigue. We also compared sleep architecture and HRV during the 30-min rest between nap chair and office chair. Brain fatigue is a proposed concept referring to stress-induced deterioration of brain function (Fujino, 1999). A health-education program prioritizing the recovery from brain fatigue, called the brain-oriented oneself-control system (BOOCS), was found to be associated with a decreased mortality from all causes in a prospective study (Hoshuyama et al., 2015). We used the term of “suspected brain fatigue” to represent high fatigue-inertia and/or low vigor-activity in the mood status.

## Methods

### Design

The study was a randomized controlled crossover trial. This study was approved by the ethics review committee of BOOCS Clinic Fukuoka and registered at the University Hospital Medical Information Network (UMIN000049314).

### Participants

Eligible subjects were male workers aged 20–64 years with suspected brain fatigue. Suspected brain fatigue was defined as having fatigue-inertia T score >60 or vigor-activity T score < 40 in the short version of the Profile of Mood Status 2 (POMS2). The POMS2 measures total mood disturbance (TMD) and 7 subscales (anger-hostility, confusion-bewilderment, depression-dejection, fatigue-inertia, tension-anxiety, vigor-activity, and friendliness) (Heuchert and McNair, 2012). The T scores of the TMD and subscales are standardized so that they have a normal distribution with a mean value of 50 points and a standard deviation (SD) of 10 points in a Japanese population as well (Yokoyama, 2015). The cutoff points of the fatigue-inertia and vigor-activity T scores corresponded to mean ± 1 × SD. Females were not included because possible changes in the hormonal status during the study period might affect the outcome parameters. Subjects were excluded if they had any of the following conditions: body mass index < 18.0 or ≥35.0 kg/m^2^, arrhythmia under medical follow-up or medication, life-limiting disease or injury, plan of changing medication or supplements during the study period, and sleep habit of excessive morning type (wake-up before 4:00 am) or night type (bed-in after 3:00 am). Furthermore, subjects were excluded if the study physician found the participation inappropriate.

### Procedures

Recruitment was carried out via poster and website announcement at the BOOCS Clinic Fukuoka in Japan. After confirmation of sex, age, job status, and literacy, potential participants underwent screening examinations for eligibility. Eligible participants were allocated to one of 2 treatment groups (office-chair rest first with crossover to nap-chair rest or nap-chair rest first with crossover to office-chair rest) in a 1:1 ratio by using a computer-generated, permuted-block randomization with block size of 4. The first experiment was done within 4 weeks after the screening examinations, and the second experiment was done 7 days after the first examination with allowance for a postponement of up to 14 days. Participants were given precautionary notes regarding the conditions which they should adhere to (Appendix 1 in Supplementary material). Specifically, the participants were asked not to change dietary and other behavioral habits during the study period. They were also requested to go to bed at 23:00–24:00 on the day prior to the study visit and to wake up at 06:00–07:00 on the day of study visit.

### Screening test

The screening examinations included questionnaire administration, anthropometric measurements, physical examinations, and laboratory measurements. The questionnaires included a lifestyle and morbidity questionnaire (sleeping habits, smoking, alcohol drinking, morbid conditions, medication, and use of supplements), the POMS2 adult short version, and the Brain Fatigue 16-item Check Sheet, which has been used in the BOOCS Clinic to collectively capture sleep problems, mental fatigue, and other psychosomatic conditions of the patients (Appendix 2 in Supplementary material). Body mass index was calculated on the basis of measured height and body weight. Percent body fat was measured by the impedance method using a commercial apparatus (In Body 770, In Body Japan, Tokyo). Venous blood was drawn after a fast of at least 5 h, and urine was collected before blood sampling. Blood cell counting, biochemical measurements, and urinalysis were done at an external laboratory (BML, Tokyo). Details of the laboratory measurements are described in Appendix 3 in Supplementary material. Plasma and erythrocyte plasmalogens were measured at the Institute of Rheological Functions of Food (Hisayama-machi, Fukuoka) according to the method described elsewhere (Mawatari et al., 2007, 2016). Plasmalogen phosphatidyl ethanolamine in plasma (mg/dL) and erythrocytes (as expressed by percentage of phospholipids) were presented here. Lowered levels of plasmalogens may be related to degenerative diseases of the brain (Hossain et al., 2020).

### Intervention

**Figure 1:**
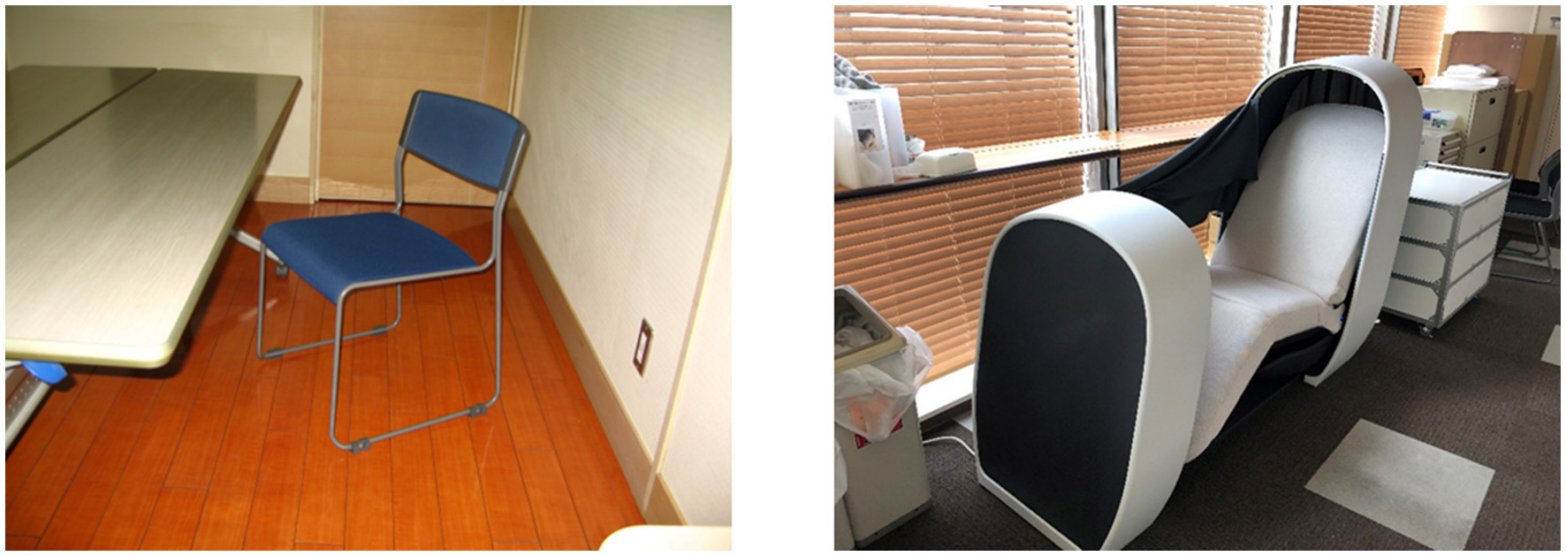
Office chair and nap chair used in intervention. The size of the nap chair **(right picture)** is 2.16 m in length, 0.74 m in width, and 1.35 m in height.

The intervention was a 30-min rest on an office chair or a nap chair as pictured in Figure 1. The nap chair has been developed for napping at the workplace, and is equipped with functions for inducing and maintaining sleep (light shielding with canopy, sound control with a headphone, temperature control with a built-in seat heater, and postural adjustment) and for waking up (sound control and postural adjustment). On sitting, angles of the seat (backrest, seat-pan, and leg-rest) are automatically adjusted to a supine posture, and the seat is covered with a canopy. Wakening is prompted with an inflation of an air bag on the back and hip, which results in a stretch of the trunk, and the seat rises from a supine position. The office-chair rest was taken on an ordinary office chair placed alongside an office table. The subjects were asked to take a rest for 30 min without any special instructions regarding sleep. The light condition was almost 0 lux in the nap-chair rest due to light shielding while the office-chair rest was taken under an ordinary office-room light condition (approximately 500 lux). The office room was air-conditioned at 25°C, and its noise level was approximately 40 dB(A). The temperature of the nap chair was adjusted to the middle of three levels, which resulted in surface temperatures of 28°C at the lower-leg part and 31°C at the backrest part in an ambient of 25°C, and a gentle wave sound of adjustable volume was provided via the headphone during the nap-chair rest.

### Time schedule on experiment days

The time schedule on the experiment days was as follows: arrival at the Clinic (12:00), lunch (12:00–12:30), attachment of apparatuses recording electroencephalogram (EEG), electrooculogram (EOG), and electrocardiogram (ECG) (12:30–12:40), before-rest outcome measurements (12:40–13:10), 30-min rest (13:10–13:40), after-rest outcome measurements (13:40–14:10), and detachment of the apparatuses (14:10). A delay of 60 min at maximum was allowed for the arrival time, and the time of the subsequent procedures was adjusted appropriately. A lunch box of the same size and menu was served at each study visit to all the participants.

The outcome measurements before and after the 30-min rest were carried out in the following order: Karolinska Sleepiness Scale questionnaire, two measurements of blood pressure after a 5-min rest in a sitting position, plethysmography, and the Uchida-Kraepelin test. The two measurements of blood pressure each before and after the 30-min rest were averaged for use in statistical analysis.

### Outcomes

The primary outcome was the after-rest change in the performance in the Uchida-Kraepelin test, which has long been used to measure cognitive performance (Kashiwagi, 1962; Seiwa, 1971; Ataka et al., 2008; Johnson et al., 2016; Saito et al., 2018; Fujino et al., 2022). The standard test forms were purchased (Japan Psychiatry Institute, Tokyo). A single test sheet contains an array of 15 lines of 116 random single-digits per line. The task is a consecutive summation of two adjacent digits per line in 1 min. Examinees perform calculation as quickly and accurately as possible and move to the next lines on examiner's cues. Numbers of attained calculations and error answers were counted per line. Percentages of correct calculations for the total 15 min and for every 5 min (first, middle, and last segments) were obtained as indices of task performance. The minute-specific pattern was also examined.

The secondary outcomes were the after-rest changes in the Karolinska Sleepiness Scale, systolic and diastolic blood pressure, 2.5-min pulse rate variability (PRV), and 15-min heart rate variability (HRV) during the Uchida-Kraepelin test. Blood pressure was measured as a marker of the autonomic nerve activity. The secondary outcomes also included sleep duration by sleep stage and HRV parameters during the 30-min rest.

The Karolinska Sleepiness Scale evaluates sleepiness on a 9-point scale (Akerstedt and Gillberg, 1990), and the Japanese version is available (Kaida et al., 2006). The ECG was recorded by using a compact Holter ECG (Cardic FLRA Holter ECG, Medilink Co., Ltd., Toyota, Aichi-ken), and the HRV analysis was done at an external laboratory (Medilink Co., Ltd., Toyota, Aichi-ken). The HRV power spectral analysis estimated the power densities of low frequency (LF) band at 0.05–0.15 Hz and high frequency (HF) band at 0.20–0.40 Hz based on an autoregressive model (Hayano et al., 1991; Sakakibara et al., 1994). Based on the reported minute-specific values of LF and HF, we calculated the average values for 3 segments of time corresponding to the 30-min rest and the Uchida-Kraepelin tests (twice of 15-min test). The HF power represents the parasympathetic activity; the LH power reflects the combined activity of sympathetic and parasympathetic nerves; and LF/HF ratio has been regarded as a sympathetic indicator (Hayano et al., 1991; Sakakibara et al., 1994). The PRV was measured by using a plethysmography (TAS9 Pulse Analyzer Plus TAS9, YKC Corporation, Tokyo). In the PRV analysis, a fast Fourier transform was used to quantify the spectral power in the LF band at 0.04–0.15 Hz and the HF band at 0.15–0.40. While the 5-min recording has usually been used in the PRV (Saito et al., 2017), the present study used a 2.5-min recording to minimize the subjects' burden. The LF and HF powers in the HRV and PRV analyses were transformed to the natural logarithmic scale because the distributions were skewed to the right side. The LF/HF was obtained based on the log-transformed LF and HF values.

EEG and EOG were recorded by using a portable recording device (Brain Wave Sensor ZA-X, Proassist, Ltd., Osaka) with disposable electrodes placed at the forehead and left mastoid (channel l) and below the outer canthus of the right eye and above the chin (channel 2). The signals were recorded at a sampling rate of 128 Hz with filters of 0.5–40 Hz for EEG and 0.5–44 Hz for EOG. The EEG/EOG recording for the 30-min rest was analyzed at an external laboratory (Rem Sleep Products Co. Ltd., Chofu-shi, Tokyo). Sleep stages during the 30-min rest were determined by 30-s epoch according to the guidelines of the American Academy of Sleep Medicine (Berry et al., 2020).

### Statistical analysis

The required number of subjects was determined to be 20. According to an unpublished information from the development team, which had included two of the authors (MI and YS) of the present study, the Uchida-Kraepelin test performance of the last one-third part improved 4 points after the nap-chair rest compared with the office-chair rest, and standard deviation (SD) of the change was approximately 5 points. The required number of subjects was estimated to be 15 for a crossover trial to detect a 4-point difference in the change with SD of 5 points, assuming a two-sided significance level of 0.05 and a statistical power of 80%. With allowance for possible dropouts, the target number of subjects was set at 20. Mean with SD and number (%) were used as descriptive statistics. Paired *t*-test was used for the comparison of the outcome measures before and after the rest separately between the treatments (nap-chair vs. office-chair). The main analysis was based on the repeated measures analysis of variance (ANOVA) with nesting for a crossover trial. The changes after the rest were used for the outcome parameters. The treatment effect, period effect, and treatment-period interaction (i.e., order effect) were always considered in the repeated measures ANOVA analysis. The effect modifications of the POMS2 fatigue-inertia and vigor-activity were examined with respect to the primary outcome measures and selected secondary outcomes. In the minute-specific analysis of the Uchida-Kraepelin test performance, *P*-values corrected for repeated measures were obtained by the Greenhouse-Geisser method. Statistical significance was declared if *P*-value was <0.05.

## Results

A total of 28 subjects received the screening examination, and 20 of them were eligible and entered the crossover trial. No dropouts occurred during the study. The study (screening and experiment) was carried out during the period from November 2022 to March 2023.

### Characteristics of the study subjects

Table 1 summarizes characteristics of the study subjects. The subjects were males aged 23–62 years, with a mean age of 43.3 years. Men with the POMS2 vigor-activity score < 40 numbered 9, and those with the fatigue-inertia score >60 numbered 15. Those having both of the conditions numbered 4. Eighteen subjects reported at least one disease under medical care. The most frequent morbid conditions were depression/dysthymia (*n* = 5), anxiety disorders (*n* = 3), insomnia (*n* = 5), sleep apnea syndrome (*n* = 3), diabetes mellitus (*n* = 5), and dyslipidemia (*n* = 3). Seven men had a habit of napping at least once per week. Among them, the weekly frequency of taking a nap ranged 1–6 days with an average of 3.4 days, and two men seemed to be regular nappers (5+ days per week). The nap length per occasion ranged 10–60 min with an average of 22 min.

**Table 1:** Characteristics of the study subjects.

**Variable**	**Mean (SD)**	**Number (%)**
Age (year)	43.3 (11.6)	
Height (cm)	172 (4.9)	
Weight (kg)	77.8 (13.2)	
Body mass index (kg/m^2^)	26.1 (3.9)	
Percent body fat (%)	24.5 (6.7)	
Plasma plasmalogen (mg/dL)	3.78 (0.99)	
Erythrocyte plasmalogen (%)	8.36 (0.77)	
Habitual sleep hours per day	6.2 (0.8)	
Brain Fatigue score	24.3 (8.6)	
**POMS2 T-score**		
Total mood disturbance	61.6 (11.7)	
Anger-hostility	51.3 (13.6)	
Confusion-bewilderment	64.5 (15.1)	
Depression-dejection	57.6 (14.7)	
Fatigue-inertia	64.9 (11.9)	
Tension-anxiety	59.0 (11.8)	
Vigor-activity	43.5 (12.0)	
Friendliness	43.8 (7.8)	
Current smoking^*^		4 (20.0)
Current alcohol use (1+ per week)		14 (70.0)
Disease under medical care		18 (90.0)
Medication		14 (70.0)
Use of supplements		12 (60.0)
Habit of napping (1+ per week)		7 (35.0)

POMS, Profile of Mood Status; SD, standard deviation.

^*^Occasional or daily smoking.

### Uchida-Kraepelin test performance

The Uchida-Kraepelin test performance before and after the rest is summarized in Table 2. There was no measurable difference in the overall and 5-min segmental performance between office chair and nap chair in either before or after the rest. The changes in the performance after the rest are shown in Table 3. The performance consistently improved after the rest. The overall 15-min score was improved after the nap-chair and office-chair rest to almost the same extent (5.9 vs. 5.5). On the other hand, the change in the performance of the middle 5 min was slightly greater after the nap-chair rest than after the office-chair rest (6.2 vs. 4.9) although the difference was not statistically significant (*P* = 0.33). No such difference was observed for either the first or last 5-min interval. A statistically significant period effect for the first 5-min performance (*P* = 0.02) indicated that the change in the performance differed in the first and second experiments, but the treatment-period interaction was not appreciable (*P* = 0.29).

**Table 2:** Performance (%) of the Uchida-Kraepelin test before and after the 30-min rest.

Time segment^*^	**Before/after rest**	**Mean (SD)**		**Paired *t*-test**
		**Office chair**	**Nap chair**	***P***
Whole 15 min	Before	46.2 (12.5)	46.8 (9.4)	0.73
	After	51.7 (11.5)	52.7 (9.6)	0.45
First 5 min	Before	46.9 (12.4)	47.6 (10.6)	0.72
	After	53.0 (12.1)	53.4 (9.6)	0.79
Middle 5 min	Before	46.1 (13.0)	46.4 (9.5)	0.89
	After	51.0 (11.5)	52.6 (9.8)	0.22
Last 5 min	Before	45.6 (12.5)	46.5 (8.8)	0.62
	After	51.0 (11.5)	52.0 (9.9)	0.46

SD, standard deviation.

^*^Time segment of the Uchida-Kraepelin test.

**Table 3:** Changes in the Uchida-Kraepelin test performance (%) after the 30-min rest: repeated measures analysis of variance with nesting.

Time segment^*^	**Mean (95% CI)**		**Treatment**	**Period**	**Interaction**
	**Office chair**	**Nap chair**	***P***	***P***	***P***
Whole 15 min	5.5 (4.1–6.8)	5.9 (4.5–7.2)	0.68	0.35	0.41
First 5 min	6.1 (4.6–7.6)	5.8 (4.3–7.4)	0.81	0.02	0.29
Middle 5 min	4.9 (2.9–6.9)	6.2 (4.3–8.2)	0.33	0.78	0.79
Last 5 min	5.4 (3.5–7.3)	5.4 (3.5–7.3)	0.98	0.80	0.42

CI, confidence interval.

^*^Time segment of the Uchida- Kraepelin test.

**Figure 2:**
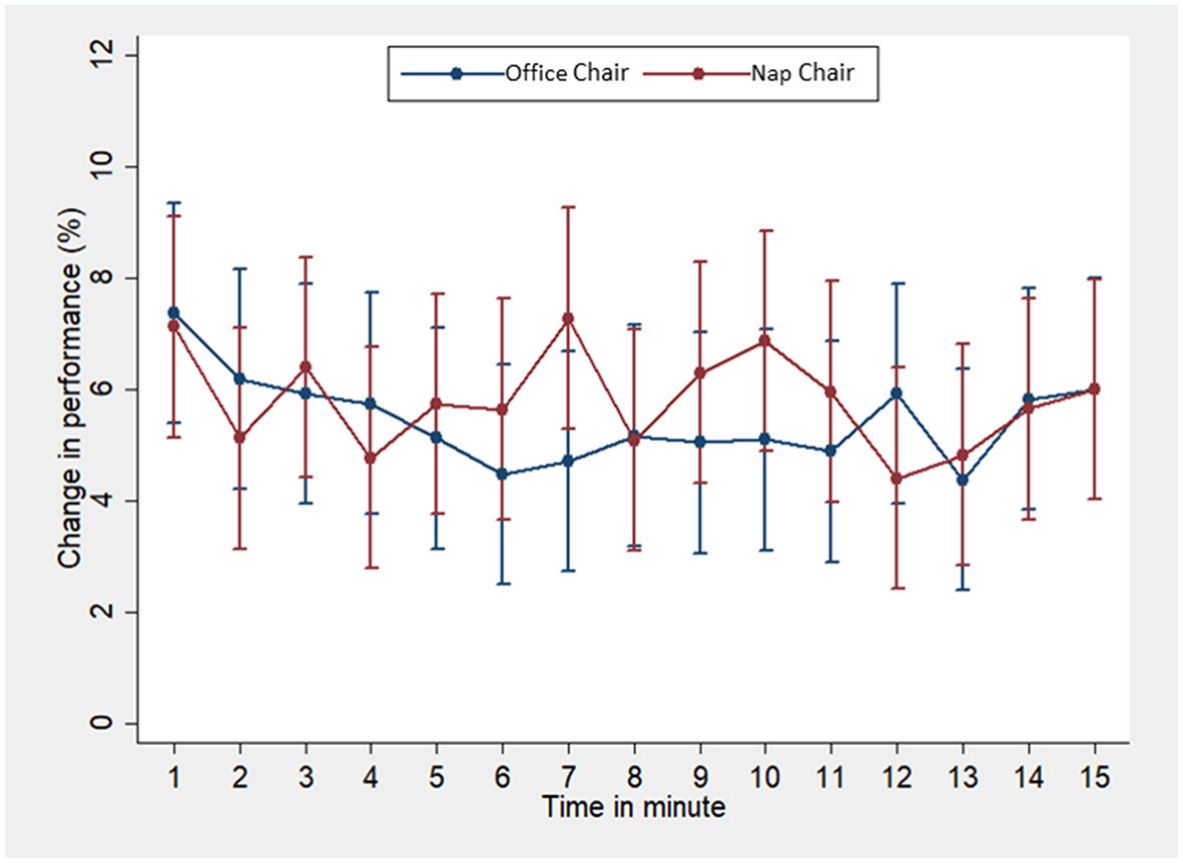
The changes in minute-specific performance in the Uchida-Kraepelin test in the office-chair rest and the nap-chair rest. The dots indicate means, and the error bars indicate 95% confidence intervals. Means and 95% confidence intervals were estimated on the basis of repeated measures ANOVA with nesting. *P* values were corrected by the Greenhouse–Geisser method: treatment *P* = 0.69, minute *P* = 0.61, and interaction *P* = 0.72.

The changes in the minute-specific performance were also examined by repeated measures ANOVA with nesting (Figure 2). The improvement in the performance after the rest tended to be greater at minutes 5–11 except minute 8 in the nap-chair rest than in the office-chair rest.

### Other outcome parameters

The Karolinska Sleepiness Scale after the rest was markedly lower in the nap-chair rest than in the office-chai-rest (*P* < 10^−3^) while the before-rest scores did not differ in the two treatments. Systolic and diastolic blood pressure, PRV parameters, and HRV parameters showed no material difference in the before-rest or after-rest measurement between office chair and nap chair (Table 4). Karolinska Sleepiness score significantly decreased after the nap-chair rest, and the between-treatment difference in the decrease was highly significant (Table 5). Systolic and diastolic blood pressure showed no appreciable changes either after the office-chair rest or after the nap-chair rest. The LF and HF powers of the PRV did not show any notable changes after the rest or between-treatment differences in the change (Table 5). The LF/HF ratio of the PRV increased significantly after the office-chair and nap-chair rest, with no difference between the treatments. Of the HRV parameters, the HF power significantly decreased after the office-chair rest, but not after the nap-chair rest, resulting in a significant difference between the two treatments. The LF/HF ratio of the HRV showed no measurable change in either office-chair rest or nap-chair rest.

**Table 4:** Secondary outcomes before and after the 30-min rest.

**Parameter (unit)**	**Before/after rest**	**Mean (SD)**		**Paired *t*-test**
		**Office chair**	**Nap chair**	***P***
Karolinska Sleepiness Scale	Before	4.6 (2.0)	4.2 (1.4)	0.32
	After	4.7 (1.7)	2.7 (1.3)	**< 10** ^**−3**^
Systolic BP (mmHg)	Before	122 (15)	124 (15)	0.30
	After	121 (15)	123 (16)	0.30
Diastolic BP (mmHg)	Before	78 (11)	80 (11)	0.18
	After	79 (11)	81 (12)	0.16
**PRV parameter** ^*^				
ln LF (ms^2^)	Before	4.50 (1.40)	4.62 (1.57)	0.75
	After	4.92 (1.25)	4.49 (1.36)	0.47
ln HF (ms^2^)	Before	4.73 (1.08)	5.03 (1.48)	0.22
	After	4.57 (1.28)	4.70 (1.53)	0.50
LF/HF	Before	0.94 (0.22)	0.92 (0.20)	0.77
	After	1.11 (0.21)	1.07 (0.24)	0.49
**HRV parameter** ^†^				
ln LF (ms^2^)	Before	5.08 (1.09)	5.17 (1.07)	0.49
	After	5.02 (0.91)	5.26 (1.04)	0.15
ln HF (ms^2^)	Before	5.06 (1.28)	4.90 (1.38)	0.41
	After	4.70 (1.14)	4.90 (1.31)	0.26
LF/HF	Before	1.03 (0.15)	1.11 (0.26)	0.10
	After	1.11 (0.23)	1.11 (0.21)	0.89

BP, blood pressure; HF, high frequency; HRV, heart rate variability; LF, low frequency; PRV, pulse rate variability.

^*^Based on 2.5-min recording.

^†^Based on 15-min recording during the Uchida-Kraepelin test. Bold values indicate *P* < 0.05.

**Table 5:** Changes in the secondary outcomes after the 30-min rest: repeated measures analysis of variance with nesting.

**Parameter**	**Mean (95% CI)**		**Treatment**	**Period**	**Interaction**
	**Office chair**	**Nap chair**	***P***	***P***	***P***
Karolinska Sleepiness Scale	0.1 (−0.4; 0.6)	−1.5 (−2.0, −1.0)	**0.0004**	0.79	0.27
Systolic BP (mmHg)	−0.3 (−3.1; 2.5)	−0.2 (−3.0; 2.7)	0.94	0.90	0.32
Diastolic BP (mmHg)	1.5 (−1.0; 4.0)	1.4 (−1.1; 3.9)	0.95	0.77	0.29
**PRV parameter** ^*^					
ln LF (ms^2^)	0.42 (−0.04; 0.88)	0.17 (−0.29; 0.63)	0.43	0.41	0.44
ln HF (ms^2^)	−0.16 (−0.50; 0.17)	−0.33 (−0.67; 0.00)	0.46	0.78	0.81
LF/HF	0.16 (0.06; 0.27)	0.15 (0.04; 0.25)	0.84	0.31	0.76
**HRV parameter** ^†^					
ln LF (ms^2^)	−0.06 (−0.17; 0.05)	0.10 (−0.01; 0.21)	**0.048**	0.52	0.25
ln HF (ms^2^)	−0.36 (−0.54; −0.17)	−0.00 (−0.19; 0.18)	**0.01**	0.33	0.44
LF/HF	0.08 (−0.01; 0.17)	0.00 (−0.09; 0.09)	0.21	0.93	0.49

BP, blood pressure; HF, high frequency; HRV, heart rate variability; LF, low frequency; PRV, pulse rate variability.

^*^Based on 2.5-min recording.

^†^Based on 15-min recording during the Uchida-Kraepelin test. Bold values indicate *P* < 0.05.

### Sleep status and HRV during the rest

During the nap-chair rest, sleep induction occurred in almost all men (n = 19), and the majority (*n* = 17) attained stage N2. During the office-chair rest, sleep induction occurred in 17 men, and 8 attained stage N2. One experienced stage N3 sleep during the nap-chair rest, and another did so during the office-chair rest. No participants experienced REM sleep during the office-chair or nap-chair rest.

The average duration of sleep was more than double during the nap-chair rest as compared with the office-chair rest with respect to all stages combined (*P* = 0.002), stage N1 (*P* = 0.005), and stage N2 (*P* = 0.008), and the between-treatment differences were highly significant (Table 6). Both LF and HF powers were significantly greater during the nap-chair rest than during the office-chair rest, and the difference in the HF power was highly significant. There was no difference in the LF/HF ratio between the office-chair rest and the nap-chair rest.

**Table 6:** Duration of sleep stages and heart rate variability parameters during the 30-min rest.

**Parameter**	**Mean (95% CI)**		**Treatment**	**Period**	**Interaction**
	**Office chair**	**Nap chair**	***P***	***P***	***P***
**Sleep stage**					
NonREM	7.6 (3.0–12.2)	19.0 (14.4–23.6)	**0.002**	0.85	0.86
N1	3.3 (1.2–5.5)	8.0 (5.8–10.1)	**0.005**	0.88	0.46
N2	4.2 (0.8–7.5)	10.9 (7.5–14.2)	**0.008**	0.99	0.74
N3	0.1 (−0.2; 0.5)	0.2 (−0.2; 0.6)	0.75	0.19	0.75
**HRV parameter**				
ln LF (ms^2^)	5.76 (5.47–6.05)	6.23 (5.94–6.52)	**0.03**	0.55	0.67
ln HF (ms^2^)	5.45 (5.24–5.67)	6.02 (5.81–6.24)	**< 10** ^**−3**^	0.42	0.83
LF/HF	1.07 (1.02–1.12)	1.05 (0.99–1.10)	0.53	0.87	0.94

HF, high frequency; HRV, heart rate variability; LF, low frequency; REM, rapid eye movement. Bold values indicate *P* < 0.05.

### Interaction with the POMS2 fatigue-inertia and vigor-activity

The effect modifications of the POMS2 fatigue-inertia and vigor-activity subscales were examined regarding the after-rest changes in the Uchida-Kraepelin test performance, Karolinska Sleepiness Scale and sleep duration during the rest (Supplementary Table 1). A suggestive interaction between the vigor-activity and treatment was noted for changes in the Uchida-Kraepelin test performance, especially of the first 5 min (*P* = 0.13) and middle 5 min (*P* = 0.22). The changes in the Uchida-Kraepelin performance according to the POMS2 vigor-activity categories are summarized in Supplementary Table 2. The between-treatment difference was the most evident for the change in the performance of the middle 5 min in the low category of the vigor-activity score (6.7 vs. 3.7) although the difference was not statistically significant (*P* = 0.12).

## Discussion

The present study did not show a favorable effect of the nap-chair rest on the Uchida-Kraepelin test performance. In healthy subjects, the performance in the Uchida-Kraepelin test generally declines with the passage of minutes but showing a last-spurt effect toward the end of the test (Kashiwagi, 1962; Fujino et al., 2022). In this regard, it would be of particular interest to examine the effect on the performance in the Uchida-Kraepelin test by time-segment. However, the present study found no appreciable difference in the improvement in the Uchida-Kraepelin test performance between the nap-chair and office-chair rest in either the overall 15-min test or any of the 5-min segments.

A long-term learning effect may have existed in the second experiment, which was carried out 1 week after the first experiment. In fact, the performance before the 30-min rest was 5 points higher in the second experiment than in the first experiment; the mean performance before the rest was 44.0 points (SD 9.7) in the first experiment and 49.1 points (SD 11.7) in the second experiment. When we examined the minute-specific performance before the rest in the first and second experiments separately, the learning effect seemed to be prominent in the early minutes of the test (Supplementary Figure 1). The time course was almost flat in the first experiment (minute *P* = 0.63) while a gradual decline was noted in the second experiment (minute *P* = 0.02). The pattern in the time course of the changes in the minute-specific performance also seemed to differ in the first and second experiments (Supplementary Figure 2). The overall treatment effect was not measurable in either experiment, but it may deserve to be mentioned that a suggestive, greater improvement in the after-rest performance was consistently observed for the nap-chair rest in the second experiment.

The nap-chair rest resulted in a sleep of 19 min on average during the 30 min of rest while the duration of sleep was < 8 min during the office-chair rest. This difference is notable even though a supine-position on the nap chair is probably more suitable for sleep than a sitting position on the office-chair. A question remains as to whether the nap chair used in the present study is superior to an ordinary bed or sofa offering a supine-position rest. It is an important finding that a sleep of stage N3 (i.e., slow-wave sleep) hardly occurred in the nap-chair rest. Arousal from a sleep of stage N3 causes sleep inertia, resulting in sleepiness, decreased alertness, and impaired task performance (Takahashi, 2003; Milner and Cote, 2009). It is noteworthy that subjective sleepiness was improved only after the nap-chair rest.

The nap-chair rest was accompanied with greater LF and HF powers, and the between-treatment difference was more evident for the HF power than for the LF power. The LF/HF ratio did not differ in the nap-chair and office-chair rest. It is known that the parasympathetic activation occurs at sleep onset and remains during sleep. The LF power as well as LF/HF ratio decreases during stage N3 sleep or slow-wave sleep, but not evidently so during stage N2 sleep (Trinder et al., 2001; Ako et al., 2003; Cellini et al., 2016; Chen et al., 2021). On the contrary, the HF power seems to increase even at stage N2 sleep (Trinder et al., 2001; Cellini et al., 2016). The present findings on the HRV parameters are thus compatible with the previous observation. The lack of the difference in the LF/HF ratio between the nap-chair rest and office-chair rest is probably linked to the observation of almost null occurrence of stage N3 sleep. The observation that the LF power was also higher in the nap-chair rest may be a chance finding, but is not a surprising finding because the LF power reflects the vagal and sympathetic activities. Overall, the nap-chair rest is likely to result in a better status of the autonomic nerve balance than the office-chair rest.

The present study objectively assessed sleep status and autonomic balance. The HRV assessment was done not only during the rest but also during the performance task. These were advantages in the present study. Limitations are noted, however. We had anticipated the learning effect in the repeated Uchida-Kraepelin test as experienced previously (Fujino et al., 2022), but did not exercise a full precaution regarding the possibility of different patterns in the before-rest minute-specific performance in the first and second experiments. The subjects may not have adapted to the test environment in the first experiment probably due to their mood problems. The sample size was not large enough to carry out the between-treatment comparison in the first and second experiments separately and to do subgroup analyses by POMS2 subscale. We did not objectively measure sleep prior to the experimental day although we asked the participants to adhere to the specification regarding the time in bed. Different sleep lengths might have affected the quality and quantity of the nap. The present study used only male participants, and the results are therefore not generalizable. Finally, the present study assessed the task performance using the Uchida-Kraepelin test. The psychomotor vigilance task (PVT) has been used for assessment of vigilant attention commonly in studies on napping and sleep (Hayashi et al., 1999a; Van Dongen et al., 2003). The PVT could be included in future studies regarding the nap-chair effects.

## Conclusion

In a crossover trial of 20 male workers with suspected brain fatigue, a 30-min rest with a nap chair did not appreciably improve the performance in the Uchida-Kraepelin test as compared with an office-chair rest. The nap-chair rest induced a substantially longer sleep of stages N1 and N2 accompanied with a parasympathetic activation, thereby resulting in a material improvement in sleepiness after the rest. Considering the recognized limitations in the present study, further large studies in both men and women are warranted regarding a potential improvement in the work performance endowed by the new nap chair.

## Data Availability

The original contributions presented in the study are included in the article/Supplementary material, further inquiries can be directed to the corresponding author.
