# Supplementary material for: Effects of a 30-min rest with a nap chair on task performance, sleepiness, and neurophysiological measures in men with suspected brain fatigue: a randomized controlled crossover trial
Source: Front Sleep. 2024 Oct 25;3:1361886. doi: 10.3389/frsle.2024.1361886 (PMC12713923; doi:10.3389/frsle.2024.1361886)
Supplement: Supplementary file 1 [file Data_Sheet_1.docx]

Supplementary Material

Effects of a 30-minute rest with a nap chair on task performance, sleepiness, and neurophysiological measures in men with suspected brain fatigue: a randomized controlled crossover trial

Minoru Fujino, Mikio Inoue, Yoshiharu Sonoda, Suminori Kono, Chikako Wakana, Shiro Mawatari, Takehiko Fujino^*^

*** Correspondence:** Takehiko Fujino: fujino-t@boocsclinic.com

**Appendix 1. Cautionary notes to participants**

The subjects were given the following specific cautions regarding screening examinations, study period, and experiment days.

1. Screening examinations

The subjects were requested to be fast for at least 5 hours on arrival at the study site and to refrain from excessive alcohol intake and exercise on the day before.

2. Study period (day of the screening examination to day of the second experiment)

The subjects were requested to live as usual; not to change dietary and other behavioral habits; not to change drugs and supplements if they are used; and to have completed acute use of drugs for cold, headache, and others until 2 days prior to the experiment day.

3. Experiment days

The subjects were requested to go to bed between 11:00 and 12:00 in the evening prior to the experiment day; to wake up between 6:00 and 7:00 in the morning of the experiment day; to take breakfast as usual on the experiment day; and to arrive at the study site at 12:00 on the experiment day with a delay of 1 hour allowable.

**Appendix 2. Brain Fatigue 16-Item Check Sheet**

The questionnaire consists of 16 questions on sleep problems, psychosomatic symptoms, mood state, mental fatigue, and others experienced over the past week (*see below*). Questions 1 to 14 have four prepared answers: almost daily (≥5 times per week), sometimes (3‒4 times per week), occasionally (1‒2 times per week), and “never”. Questions 15 and 16 have 4 options of quartile percentages (<25%, 25‒49%, 50‒74%, and ≥75%). Score 0, 1, 2, or 3 (worst) is assigned to each answer, and the Brain Fatigue score is obtained by totaling the scores and ranges from 0 to 48. Questions 1 to 11 are available at the website of BOOCS Clinic Fukuoka (https://www.boocsclinic.com/fukuoka/check/).

1. Do you awake during the night and/or awake earlier than desired in the morning?
2. Do you have difficulty in falling asleep?
3. Do you eat for routine and/or without appetite?
4. Do you have constipation?
5. Do you feel exhausted without physical exertion?
6. Do you feel gloomy and depressed?
7. Do you feel hopeless?
8. Do you feel unsettled in thought?
9. Do you feel restless and nervous?
10. Do you feel uneasy and anxious?
11. Do you think that you are a worthless person?
12. Do you have headache, shoulder stiffness or lumbago?
13. Do you feel that you have many things to do or worry about?
14. Are you lively and energetic?
15. What percentages have you achieved of your main work?
16. What is your present work capability in percentage as compared with the best?

**Appendix 3. Laboratory test items**

*Blood cell count:* red blood cell count, hemoglobin, hematocrit, white blood cell count, and platelet count.

*Blood biochemical measurements:* serum values of total protein, total bilirubin, aspartate aminotransferase (AST), alanine aminotransferase (ALT), gamma-glutamyltransferase (GGT), alkaline phosphatase (ALP), lactate dehydrogenase (LDH), creatine phosphokinase (CPK), total cholesterol, high-density lipoprotein (HDL) cholesterol, low-density lipoprotein (LDL) cholesterol, triglycerides, uric acid, blood urea nitrogen (BUN), creatinine; plasma glucose; and hemoglobin A1c.

*Qualitative urinalysis:* sugar, protein, and occult blood.

**Supplementary Table 1.** *P* values for the effect modifications of POMS2 fatigue-inertia and vigor-activity on selected outcome parameters

| Outcome parameter | Fatigue-inertia | Vigor-activity |
| --- | --- | --- |
| Change in the Uchida-Kraepelin test performance |  |  |
| Whole 15minutes | 0.40 | 0.23 |
| First 5 minutes | 0.53 | 0.13 |
| Middle 5 minutes | 0.35 | 0.22 |
| Last 5 minutes | 0.79 | 0.93 |
| Change in the Karolinska Sleepiness Scale | 0.79 | 0.42 |
| Duration of sleep during the 30-minute rest |  |  |
| Non-REM | 0.92 | 0.96 |
| Stage N1 and N2 | 0.94 | 0.87 |

POMS: Profile of Mood Status.

Based on repeated measurements analysis of variance with nesting, in which the fatigue-inertia and vigor-activity scores were classified into and low and high categories using the median as cutoff point.

**Supplementary Table 2.** Effect modification of POMS2 vigor-activity score on the changes in the Uchida-Kraepelin test performance (%) after the 30-minute rest

| Time segment | POMS2 | Mean (95% CI) |  | *P* |
| --- | --- | --- | --- | --- |
|  | vigor-activity | Office chair | Nap chair |  |
| Whole 15minutes | Low | 4.3 (2.4–6.1) | 5.7 (3.9–7.6) | 0.25 |
|  | High | 6.7 (4.8–8.5) | 6.0 (4.1–7.8) | 0.57 |
| First 5 minutes | Low | 4.3 (2.0–6.7) | 5.9 (3.5–8.2) | 0.35 |
|  | High | 7.8 (5.5–10.2) | 5.8 (3.5–8.2) | 0.22 |
| Middle 5 minutes | Low | 3.7 (1.0–6.4) | 6.7 (4.0–9.4) | 0.12 |
|  | High | 6.1 (3.4–8.8) | 5.8 (3.1–8.5) | 0.88 |
| Last 5 minutes | Low | 4.7 (2.0–7.4) | 4.6 (1.9–7.3) | 0.97 |
|  | High | 6.1 (3.4–8.8) | 6.3 (3.6–9.0) | 0.94 |

POMS: Profile of Mood Status.

Based on repeated measurements analysis of variance with nesting, in which the vigor-activity score was classified into and low and high categories using the median as cutoff point. *P* values represent treatment effects in each category.

**Supplementary Figure 1.**

Minute-specific performance in the Uchida-Kraepelin test before the 30-minute rest in the first experiment (left panel) and second experiment (right panel). Means (indicated by dots) and 95% confidence intervals (vertical lines) were estimated on the basis of repeated measures ANOVA with minute as a variable of repeated measures. *P* values were corrected by the Greenhouse–Geisser method: *P* for minute was 0.63 in the first experiment and 0.02 in the second experiment.

**
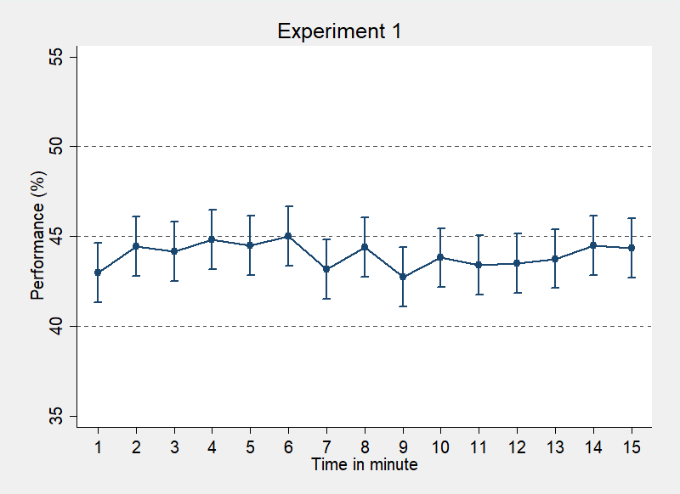

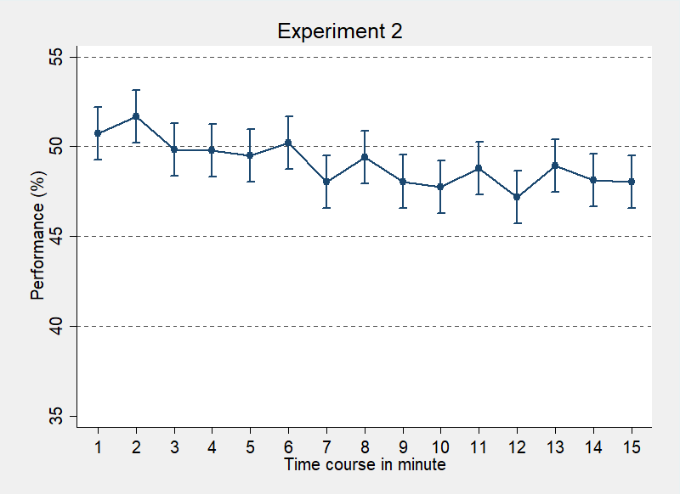
**

**Supplementary Figure 2.**

The changes in minute-specific performance in the Uchida-Kraepelin test in the first experiment (left panel) and second experiment (right panel). Means (indicated by dots) and 95% confidence intervals (vertical lines) were estimated on the basis of repeated measures ANOVA with splitting. *P* values were corrected by the Greenhouse–Geisser method: treatment *P* = 0.59, minute *P* = 0.11, and interaction *P* = 0.62 in the first experiment; treatment *P* = 0.39, minute *P* = 0.51, and interaction *P* = 0.98 in the second experiment.

**
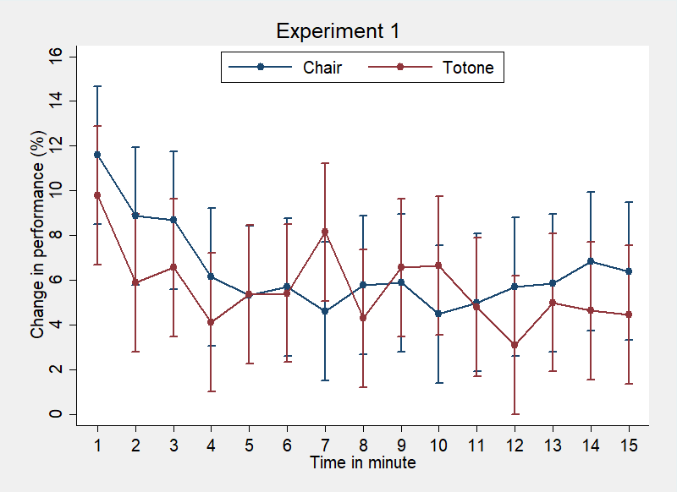

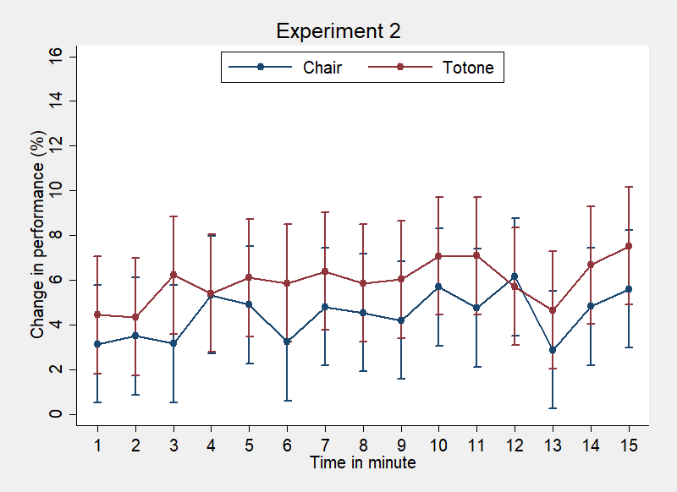
**

Office Chair

Nap Chair

Office Chair

Nap Chair
